# Therapeutic strategies to ameliorate mitochondrial oxidative stress in ischaemia-reperfusion injury: A narrative review

**DOI:** 10.1042/CS20242074

**Published:** 2025-02-03

**Authors:** Khalid Alotaibi, Nishkantha Arulkumaran, Alex Dyson, Mervyn Singer

**Affiliations:** 1 Bloomsbury Institute of Intensive Care Medicine, Division of Medicine, University College London, London, U.K; 2 King Faisal Specialist Hospital & Research Centre, Riyadh, Saudi Arabia; 3 Centre for Pharmaceutical Medicine Research, Institute of Pharmaceutical Science, King’s College London, London, U.K

**Keywords:** antioxidant, ischaemia-reperfusion injury, mitochondria, mROS, oxidative stress

## Abstract

Mitochondrial reactive oxygen species (mROS) play a crucial physiological role in intracellular signalling. However, high levels of ROS can overwhelm antioxidant defences and lead to detrimental modifications in protein, lipid and DNA structure and function. Ischaemia-reperfusion injury is a multifaceted pathological state characterised by excessive production of mROS. There is a significant clinical need for therapies mitigating mitochondrial oxidative stress. To date, a variety of strategies have been investigated, ranging from enhancing antioxidant reserve capacity to metabolism reduction. While success has been achieved in non-clinical models, no intervention has yet successfully transitioned into routine clinical practice. In this article, we explore the different strategies investigated and discuss the possible reasons for the lack of translation.

## Introduction

The optimal therapeutic intervention for salvaging ischaemic tissue entails the timely restoration of blood flow – ‘reperfusion’. However, reperfusion itself induces paradoxical damage, resulting in the condition termed *‘*ischaemia-reperfusion injury (IRI)’. The concept originated nearly six decades ago when Jennings et al. reported an increase in myocardial infarct size upon reperfusion [1]. In the 1970s, Hearse et al. observed that the reintroduction of oxygen to deprived tissues caused a distinct injury, separate from the initial ischaemic insult [2].

**Figure 1:**
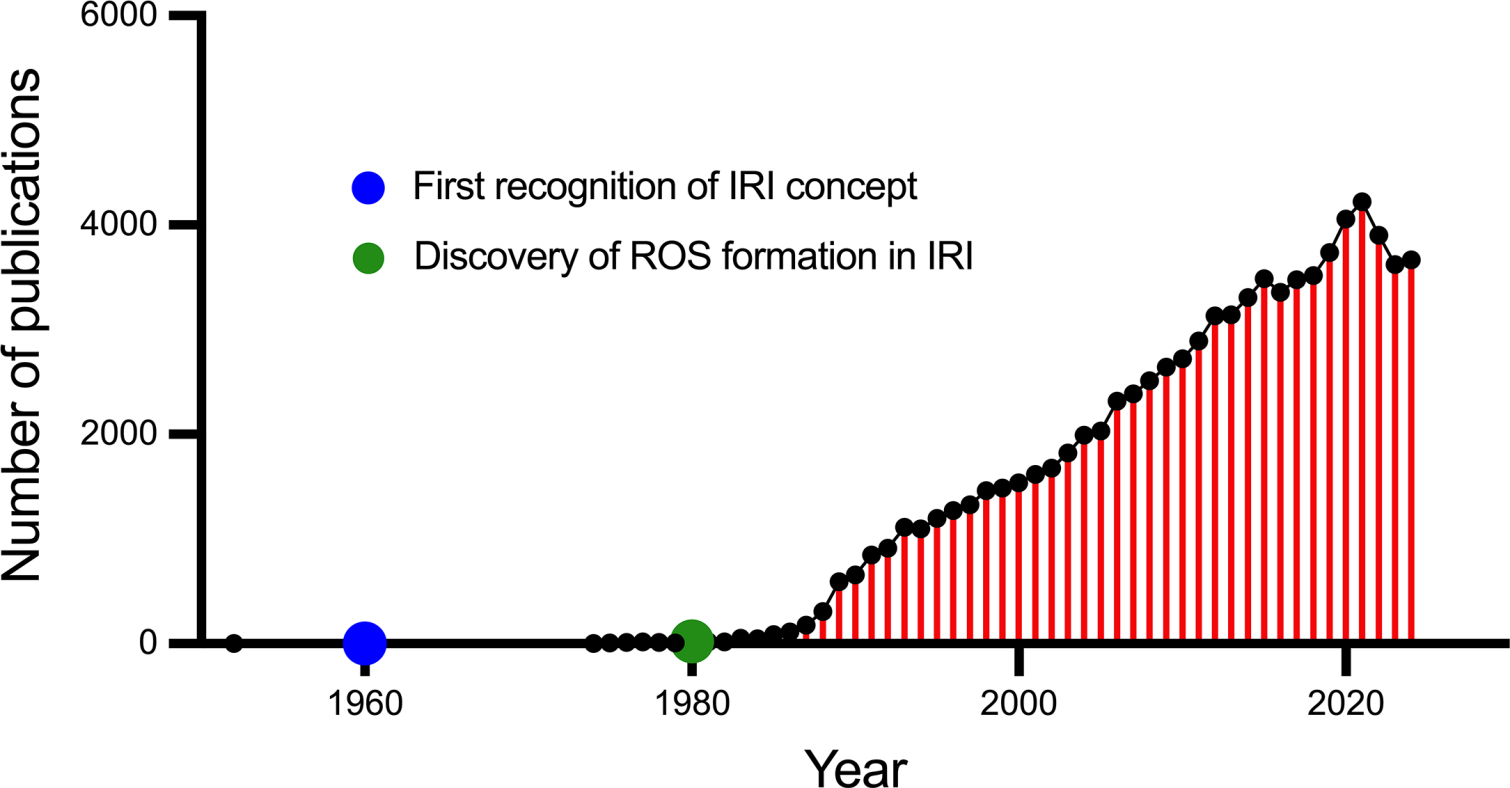
Publications by year related to ischaemia-reperfusion injury. The search on this database (with data extraction) was conducted on December 22, 2024. IRI, ischaemia-reperfusion injury,; ROS:, reactive oxygen species.

Since the inception of the IRI concept, a rapidly growing body of literature has investigated this pathological phenomenon and its association with various medical procedures including, but not limited to, cardiopulmonary resuscitation (CPR), revascularisation of blocked arteries (e.g. stroke and myocardial infarction) and organ transplantation (Figure 1). In the early 1980s, the concept of ‘oxygen-derived free radicals’ – now termed reactive oxygen species (ROS) – was proposed as a major driving force behind the damage caused by IRI [3]. This premise was rapidly embraced, as the damage induced by IRI was clearly apparent when molecular oxygen was introduced [3]. Reintroduction of oxygen to oxygen-starved tissues created a mismatch between the rate of ROS production and removal [4]. As ROS are formed predominantly within mitochondria, many subsequent studies have targeted mitochondrial pathology and protection strategies.

This article considers the physiological significance of mitochondrial reactive oxygen species (mROS) in health and their contribution to IRI. The diverse therapeutic approaches aimed at ameliorating mitochondrial oxidative stress in the context of IRI are subsequently examined.

## Mechanisms of mROS formation in health

Mitochondria are the predominant source of ROS, with multiple pathways identified for ROS production [5,6]. Production primarily occurs during oxidative phosphorylation (OXPHOS) at complexes I and III of the electron transport chain (ETC) [6,7]. This occurs in both health and disease states, predominating in the latter in which endogenous antioxidant defences are frequently overwhelmed.

OXPHOS involves a series of oxidation and reduction reactions resulting in the generation of adenosine triphosphate (ATP). These reactions are catalysed by a series of mitochondrial enzymes (complexes I–IV), collectively forming the ETC [8]. Briefly, NADH and FADH_2_, generated predominantly by the citric acid (Krebs’) cycle, donate electrons to complexes I and II, respectively. Ubiquinone receives electrons predominantly from complexes I and II and transfers them to complex III [8]. Complex III contains two ubiquinone sites (Q_o_, Q_i_). At the Q_o_ site, ubiquinol (the reduced form of ubiquinone) passes a single electron to cytochrome c (Cyt c) and the other to the Q_i_ site [7,8]. Reduced Cyt c then shuttles electrons to complex IV, which consists of four metal-based groups facilitating electron transfer to oxygen, the terminal electron acceptor [7,9,10].

**Figure 2:**
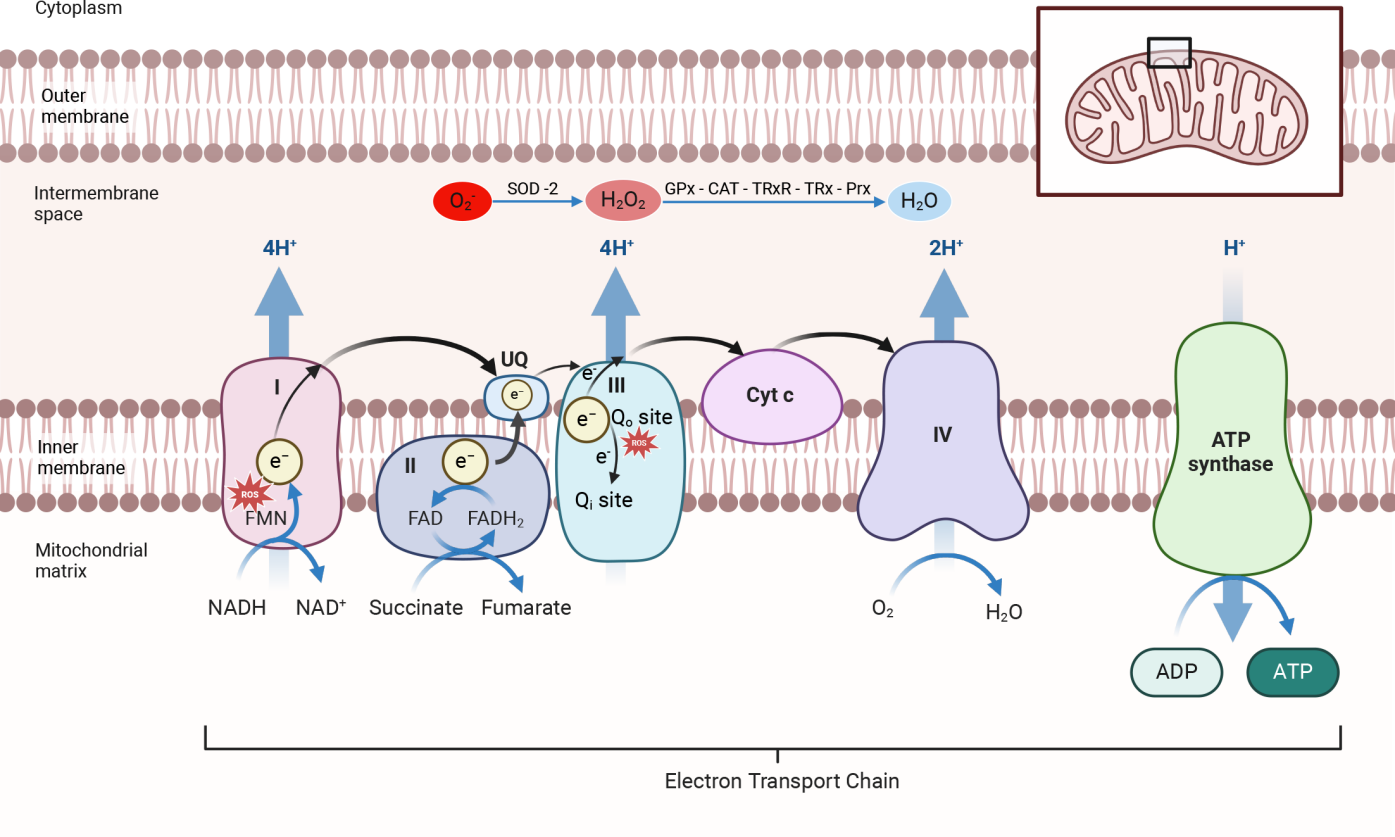
Mitochondrial physiology and mechanisms of mROS production in health. During normal electron transport chain (ETC) flow, NADH and FADH_2_ donate electrons to complexes I and II, respectively. Electrons pass down the chain with oxygen being the terminal electron acceptor at complex IV. In doing so, protons are pumped into the intermembrane space via complexes I, III and IV creating an electrochemical gradient. This energy gradient is used by complex V (ATP synthase) to phosphorylate adenosine diphosphate (ADP) to adenosine triphosphate (ATP), which is then used to fuel cellular metabolism. As electrons are carried through the ETC, a small fraction prematurely binds to oxygen, forming superoxide (O_2_^•-^). Red stars indicate the most common sources of mROS in health. Mitochondria have strong antioxidant systems that rapidly scavenge mROS to less potent ROS and then to water. CAT, catalase; e^-^, electron; FMN, flavin mononucleotide; GPx, glutathione peroxidase; H_2_O, water; H_2_O_2_, hydrogen peroxide; O_2_, oxygen; Prx, peroxiredoxin; ROS, reactive oxygen species; SOD-2, superoxide dismutase-2; TRx, thioredoxin; TRxR, thioredoxin reductase; UQ, ubiquinone.

As electrons flow through the respiratory chain, complexes I, III and IV induce reactions leading to proton movement from the mitochondrial matrix to the intermembrane space, thereby creating an electrochemical gradient [7,11]. This gradient drives a conformational rotation of complex V (ATP synthase), enabling phosphorylation of adenosine diphosphate (ADP) to ATP [12]. However, as electrons are carried through the ETC, they may prematurely bind to oxygen, forming superoxide (O_2_^•-^) [7,13]. While several sites within mitochondria produce ROS in health, complexes I and III are the primary sources [14–17]. Figure 2 depicts mechanisms of ROS formation within the ETC in health.

## The physiological role of mROS in health

Physiological mROS production accounts for approximately 1–2% of oxygen consumed by mitochondria in health. mROS play an integral role in intracellular signalling. They are critical mediators within cell differentiation pathways [18–21] and play a role in autophagy and mitophagy [22,23]. mROS play a significant role in adaptation pathways to hypoxia [24,25] and are also required for various functional responses in immune cells including inflammasome activation and phagocytic killing of pathogenic bacteria [26–28].

## Regulation of mROS (endogenous)

### Antioxidants

Mitochondria are equipped with powerful antioxidant systems that rapidly scavenge mROS. Superoxide dismutase (SOD)-2 is exclusively localised within the mitochondrial matrix where it catalyses dismutation of O_2_^•-^ to hydrogen peroxide (H_2_O_2_) [29]. H_2_O_2_ is relatively stable and less harmful compared with O_2_^•-^, but it too is an oxidant. Numerous mitochondrial antioxidant systems actively contribute to the conversion of H_2_O_2_ into water and oxygen, including catalase (CAT) and the thioredoxin system (thioredoxin reductase (TRxR), thioredoxin (TRx) and peroxiredoxin (Prx)) [30,31] (Figure 2).

### Uncoupling proteins

Uncoupling proteins (UCPs) are a five-member family (UCP1-5) of mitochondrial carrier proteins. UCP-1, located within brown adipose tissue, enables a regulated leak of protons across the inner mitochondrial membrane, thereby diverting energy from ATP synthesis to thermogenesis [32]. UCP-2 and UCP-3 are present at much lower abundances than UCP1 [33]. UCP-2, present in multiple tissues, is up-regulated by an increase in H_2_O_2_, while UCP-3 is overexpressed in skeletal muscle triggered by an increase in H_2_O_2_ and circulating free fatty acids [32,34,35]. UCPs inhibit mROS production via a reduction in the mitochondrial membrane potential [36,37]. UCP-4 and UCP-5 may be involved in thermoregulation within the brain and also protect against oxidative stress [38].

## Mechanisms of mROS formation in IRI

**Figure 3:**
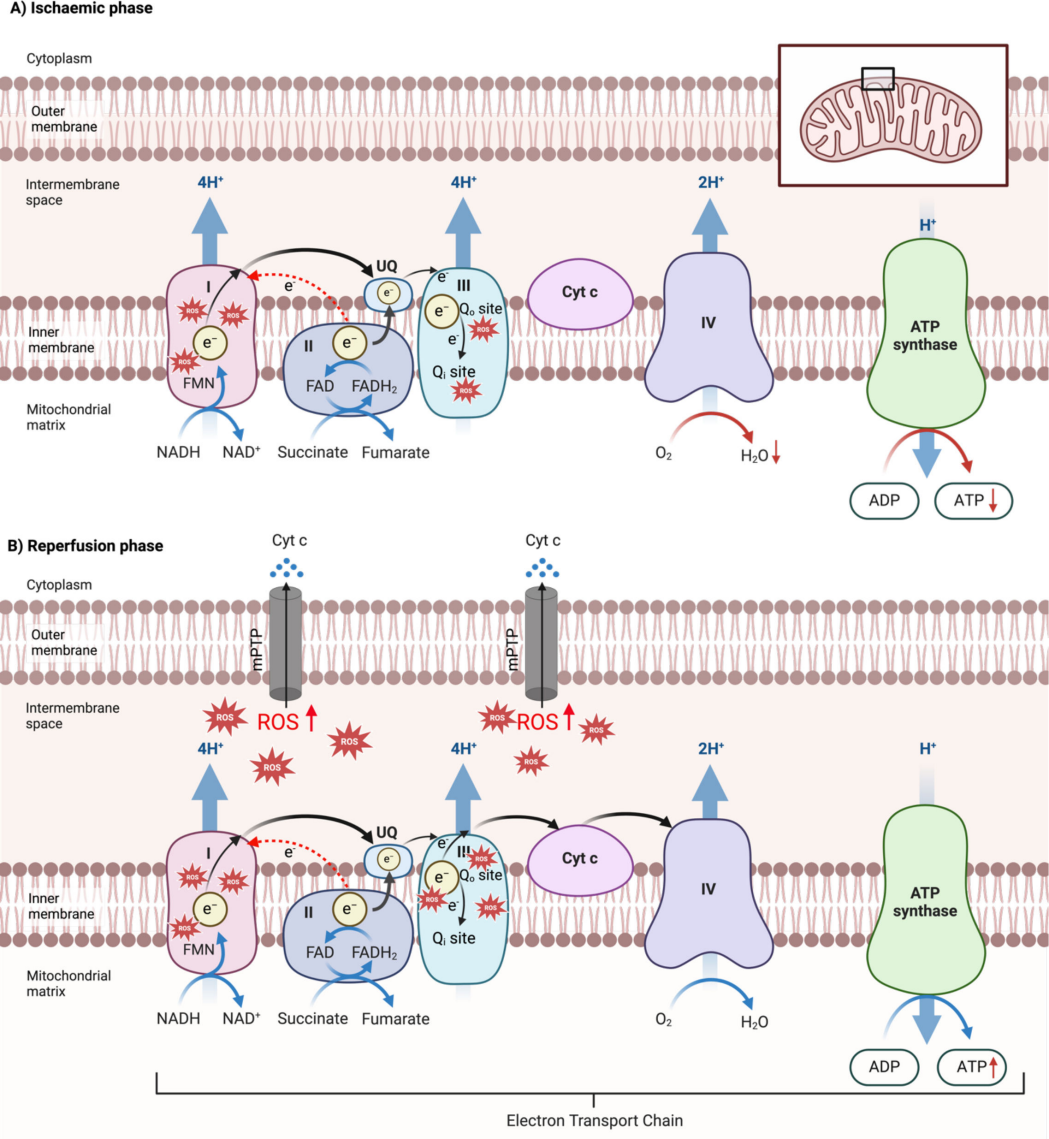
Mechanisms of mROS formation during ischaemia and reperfusion phases. (**A) Ischaemia phase**. During hypoxia (ischaemia), electron flow down the electron transport chain (ETC) falls. Electrons accumulating at complex II lead to reverse electron flow towards complex I (indicated by red-dotted arrows) with increased ROS production and decreased ATP synthesis. Reduced electron flow also promotes ROS generation at complex III, particularly at the Q_i_ and Q_o_ sites within the Q cycle. These events cause the accumulation of mitochondrial metabolites, setting the stage for an ‘oxidative burst’ upon reperfusion/reoxygenation. (**B) Reperfusion phase**. Upon reoxygenation, the substrate-driven ETC resumes operation at high capacity, generating more ATP as well as more mitochondrial ROS (mROS). This also forces electrons to flow in reverse from complex II to complex I (again indicated by red-dotted arrows). Red stars mark common sources (complexes I and III) of mROS during reperfusion. The increase in mitochondrial oxidative stress triggers the opening of the mitochondrial permeability transition pore (mPTP) and release of cytochrome Cc (Cyt c), trigerring cell death pathways. e, electron; H_2_O, water; O_2_, oxygen; ROS, reactive oxygen species; UQ, ubiquinone.

The pathophysiology of IRI can be broadly divided into ischaemic and reperfusion phases [39]. During the ischaemic phase, oxygen depletion results in the slowing of electron flow through the ECT, a fall in mitochondrial membrane potential and a reduction in ATP production [40,41]. The cell reacts by increasing glycolytic (anaerobic) respiration to partially compensate for the decrease in ATP generated by OXPHOS. However, with ischaemia, the rate of ATP production remains insufficient to meet cellular metabolic demands. The decreased flow through the ECT facilitates mROS formation at complex III within the Q cycle and the accumulation of metabolites including succinate, resulting in reverse electron flow from complex II to complex I with increased mROS production at the flavin mononucleotide site ( Figure 3-A) [42,43].

Tissue hypoxia sets the scene for excessive mROS formation on reoxygenation [44–46]. The more the mitochondrial metabolites that accumulate during the ischaemic phase, the greater the mROS formation that occurs at reperfusion [42,46,47]. While the extent of pathological damage during the ischaemic interval varies among tissues, it correlates with the duration of ischaemia [48]. At reperfusion, with restoration of oxygen, electron flow through the ETC is significantly accelerated. Mitochondrial membrane potential increases above normal, driving both ATP and mROS production [46]. Succinate accumulated during ischaemia is restored to pre-ischaemic levels within 5 minutes [42]. There is an increased counter-directional movement of electrons from complex II to complex I, resulting in greatly increased ROS production (Figure 3B). Antioxidant defences are overwhelmed, allowing excess mROS to cause damage to its mitochondrial host, other cellular organelles and the plasma membrane. Mitochondrial oxidative stress also opens the mitochondrial permeability transition pore (mPTP) with the release of mitochondrial molecules (e.g. Cyt c and mitochondrial DNA) into the cytosol that, in turn, triggers various cell death pathways [49,50]. Released mitochondrial molecules also act as damage-associated molecular patterns, triggering a systemic inflammatory response [51]. mROS also directly induces the assembly and activation of the NLRP3 inflammasome complex [52,53] that further amplifies the inflammatory response and the pathological progression of IRI. However, as a protective mechanism to mitigate oxidative stress, mitophagy is thereby, activated to selectively degrade damaged mitochondria [54,55].

**Figure 4:**
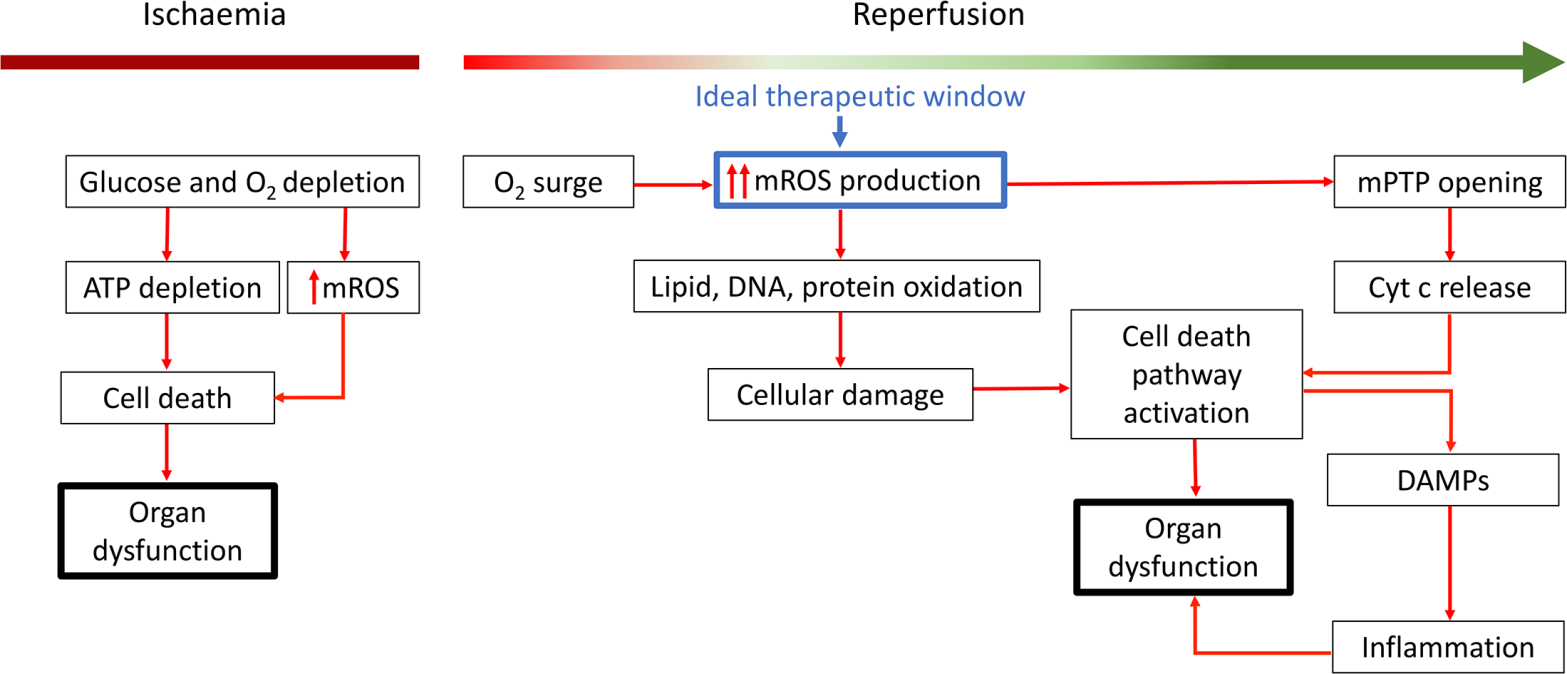
Mitochondrial reactive oxygen species (mROS) production pathways during IRI and associated damage mechanisms. ATP: adenosine triphosphate, Cyt c: cytochrome c, DAMPs: damage-associated molecular patterns, O_2_: oxygen.

A significant surge in mROS production initially occurs within minutes of reoxygenation, marking the hyperacute phase [56–59]. This temporal window presents a crucial opportunity for targeted interventions. While enhancing mitochondrial antioxidants is generally considered a protective strategy, incomplete downstream scavenging may still permit some mROS-mediated damage. In contrast, directly modulating metabolism to reduce mROS production is more likely to mitigate upstream effects by lowering mROS formation, thereby preventing secondary damage such as the activation of apoptotic or inflammatory pathways. Figure 4 illustrates cellular pathways activated by mROS elevation during IRI and highlights the therapeutic window for intervention.

### Other mechanisms underlying IRI – calcium overload

During the ischaemic phase, anaerobic respiration increases. Intracellular H^+^ accumulation leads to decreased intracellular pH and increased sodium influx through the sodium/hydrogen exchanger (NHE) [60]. Excessive intracellular sodium promotes sodium excretion and calcium uptake via the sodium/calcium exchanger (NCE), resulting in significant intracellular calcium accumulation and subsequent calcium overload. Upon re-oxygenation, extracellular pH increases while intracellular pH remains acidic [60]. This pH gradient facilitates the extrusion of H^+^ from the cell in exchange for sodium [61]. The increased cytosolic sodium can be extruded by Na^+^-K^+^ -ATPase or the NCE in exchange for potassium and calcium, respectively, thereby raising intracellular calcium levels [61]. Direct H^+^/Ca^2+^ exchange also contributes to calcium overload [60]. Elevated intracellular calcium activates further calcium-induced calcium release from the sarcoplasmic/endoplasmic reticulum into the cytosol [62–64]. In addition, excess mROS can damage cell membrane integrity, increasing permeability and extracellular Ca^2+^ influx leading to hypercontracture [60,65]. mROS also damage the sarcoplasmic/endoplasmic reticulum membrane, further exacerbating intracellular calcium overload [60,62]. Excessive intracellular calcium enters mitochondria impairing ATP production, activating caspases and calpains, and triggering apoptotic cell death pathways [66].

## Therapeutic strategies trialled to mitigate mitochondrial oxidative stress

### Inducing mild-to-moderate oxidative stress

mROS at supraphysiological, but non-toxic, concentrations participate in signalling processes that play a protective role [67–69]. This has been applied therapeutically by inducing mild-to-moderate oxidative stress using approaches such as ischaemic pre-conditioning (IPC) and MitoParaquat.

#### Direct and remote ischaemic pre-conditioning

IPC involves cycles of transient occlusion of blood flow before reperfusion [70]. This strategy was first conceived by Mutty et al. in 1986, who demonstrated its cardioprotective effects [71]. Various techniques have been employed, including proximal or distal methods. Proximal IPC typically involves local occlusion–reperfusion of the affected organ, while distal IPC often uses an inflated blood pressure cuff applied to a limb. Clinical studies have, however, yielded variable results [72], and the optimal technique for IPC remains to be definitively established.

IPC was proposed to enhance resilience of ischaemic tissues to the deleterious effects of ‘second hit’ reperfusion [73]. By inducing mild oxidative stress during the ischaemic phase, IPC initiates signalling pathways to up-regulate the production of mitochondrial antioxidant enzymes [73–77]. Animal models demonstrate increased antioxidant activity in myocardial and cerebral tissues following IPC [74–76]. Of note, administration of antioxidants attenuated the protective effects of IPC [78–80].

Remote ischaemic pre-conditioning (RIPC) utilises transient episodes of ischaemia and reperfusion usually induced by intermittent inflation of an arm blood pressure cuff to protect a distant organ [81]. Previous studies in cardiac surgery suggested cardioprotective effects, including reduced postoperative troponin-I levels [82,83]. However, three large randomised controlled trials (RCTs) investigating RIPC in elective cardiac surgery and before primary percutaneous coronary intervention (PCI) failed to demonstrate clinical benefit [84–86]. Large RCTs investigating the impact of RIPC prior to revascularisation of acute ischaemic stroke have also revealed conflicting results [87–89].

#### Ischaemic post-conditioning

Ischaemic post-conditioning (IPostC) involves brief ischaemic episodes during early reperfusion. It has been applied both proximally and distally to IRI sites [70]. In dogs, cycles of coronary occlusion–reperfusion during early myocardial reperfusion reduced infarct size similar to IPC [56]. Small proof-of-concept clinical trials suggested that combining PCI with IPostC may reduce infarct size [90–93]; however, magnetic resonance imaging-based studies yielded mixed results [94–96]. The largest RCT, involving 1234 patients with ST-elevation myocardial infarction (STEMI) undergoing conventional PCI with or without IPostC (four cycles of 30 second balloon occlusions and reperfusion), found no effect on either all-cause mortality or hospitalisation for heart failure [97]. Studies utilising both RIPC and IPostC in elective cardiac surgery and during PCI for acute myocardial infarction also found no impact on clinical outcomes [98,99].

#### MitoParaquat

MitoParaquat is a selective mitochondrial superoxide generator that moderately elevates mROS in anoxia/reoxygenation and cardiac IRI models [100]. Lower doses were protective, while higher doses disrupted Ca^2+^ homeostasis and mitochondrial function, leading to cell dysfunction and death. The suggested mechanism is the preservation of mPTP integrity [100].

### Metabolism reduction

#### Targeted Temperature Control

Targeted temperature control (TTC), also known as therapeutic hypothermia or targeted temperature management, aims to reduce metabolism by lowering core temperature to between 32°C and 34°C. In animal models, TTC decreases cerebral oxygen consumption, and mROS production, andleading to improved clinical outcomes in the models of circulatory arrest [101–105].

TTC has been investigated clinically in numerous IRI conditions including comatose patients surviving cardiac arrest, MI, stroke, and neonates born with hypoxic-ischaemic encephalopathy (HIE). However, as described below, these clinical trials have been disappointing and have not translated into routine clinical practice in most IRI conditions. Optimal duration [106] and depth of cooling remain uncertain [107,108].

##### Targeted Temperature Control in cardiac arrest

In 2002, two small RCTs found long-term neurofunctional benefits from TTC in out-of-hospital cardiac arrest (OHCA) patients [109,110]. Consequently, TTC was widely adopted,; however, two subsequent large RCTs failed to show any difference, either for survival or neurological outcomes [111,112]. Of note, the target temperature was only reached at around approximately 8 hours after the initial insult (Table 1); this delay may explain the negative results. Experimental data suggest a therapeutic window of up to 4 hours for TTC [113]. A recent study did show improved neurological outcomes, but not survival, in patients with non-shockable rhythms undergoing TTC [114].

**Table 1:** Summary of the major RCTs testing Targeted Temperature Control in ischaemia-reperfusion injury conditions.

Study	Design	Sample size	Condition	Temperatures(°C)	Duration (hours)	Time to TT (hours)	Improved survival	Improved neurological function
Hachimi-Idrissi et al. 2001 [115]	P-RCT	30	OHCA	34 vs. N	4	7	No	No
Bernard et al. 2002 [110]	Qm-rct	77	OHCA	33 vs. N	12	8	Yes	Yes
HACA 2002 [109]	M-RCT	275	OHCA	33 ± 1vs. N	24	9	No	Yes
Hachimi-Idrissi et al. 2005 [116]	S-RCT	33	OHCA	33 vs. N	4	NR	No	No
Hachimi-Idrissi et al. 2005 [116]	S-RCT	28	OHCA	33 vs. N	24	NR	No	No
TTM-1 Trial 2013 [111]	M-RCT	950	OHCA	33 vs 36	28	8	No	No
Lascarrou et al. 2019 [114]	M-RCT	584	CA	33 vs. < 37.8	24	9	No	Yes
TTM-2 Trial 2021 [112]	M-RCT	1851	OHCA	33 ± 0.5 vs 37 ± 0.5	28	8	No	No
Dixon et al. 2002[117]	M-RCT	42	MI	33 vs. N	4	0.5	No	NA
Götberg et al. 2010 [118]	S-RCT	20	MI	33 vs. N	3	0.5	No	NA
Erlinge et al. 2014 [119]	M-RCT	120	MI	33 vs. N	1	0.5	No	NA
Nichol et al. 2015 [120]	M-RCT	54	MI	32.5–34.9 vs. N	3	0.3	No	NA
Noc et al. 2021 [121]	M-RCT	111	MI	32 vs. N	3	1	No	NA
Els et al. 2006 [122]	S-RCT	25	MCAO	35 vs. N	48	3	No	No
Neugebauer et al. 2019 [123]	M-RCT	50	MCAO	33 ± 1 vs N	72	24	No	No
Gluckman et al. 2005 [124]	M-RCT	218	Hi.e.	34–35 vs. 37 ± 0.2	72	6 >	No	Yes^1^
Shankaran et al. 2005 [125]	M-RCT	205	Hi.e.	33.5 vs 36.5-37	72	6 >	No	Yes^1^
Azzopardi et al. 2009 [126]	M-RCT	325	Hi.e.	33–34 vs. 37 ± 0.2	72	6 >	No	Yes
Simbruner et al. 2010 [127]	M-RCT	129	Hi.e.	33–34 vs. 37 ± 0.5	72	6.2	Yes	Yes
Azzopardi et al. 2014 [128]	M-RCT	325	Hi.e.	33–34 vs. N	72	NR	No	Yes

^1^infants with moderate HIE

CA, cardiac arrest (in-hospital and out-of-hospital). HIE, hypoxic-ischaemic encephalopathy. MCAO, middle cerebral artery occlusion. MI, myocardial infarction. M-RCT, multi-centre randomised controlled trial. N, normothermia. NA, not applicable. NR, not reported. OHCA, out-of-hospital cardiac arrest. P-RCT, pilot randomised controlled trial. QM-RCT, quasi-multi-centre randomised controlled trial. S-RCT, single-centre randomised controlled trial.

##### Targeted Temperature Control in myocardial infarction

TTC in animal models resulted in reduced infarct size and coronary microvascular injury if the target temperature was achieved before reperfusion [129–131]. Small RCTs in STEMI patients, however, failed to demonstrate significant infarct size reduction [117,119–121]. Again, this may be due to technical challenges in achieving rapid, effective cooling in clinical settings. Both cold saline infusion (0.6–2 litres) [118,120] and endovascular cooling [117] were both able to reduce core temperature to aroundapproximately 34.5°C at the time of PCI, but failed to reach the target of 32-33°C. Moreover, these procedures increased door-to-balloon time, potentially causing detriment through a prolonged ischaemia time [120,121].

##### Targeted Temperature Control in ischaemic stroke

Non-clinical data suggest that TTC is most effective when initiated early after vessel occlusion, at a moderate cooling level (32-34°C) and maintained for over 48 hours [132–134]. TTC combined with hemicraniectomy has been studied in patients with malignant ischaemic stroke. A pilot study using an intravascular cooling catheter was able to safely maintain core temperature at 35°C for 48 hours [122]. A later study induced TTC using cooled saline and maintained a core temperature of 33 ± 1.0°C for three days with intravascular or surface cooling [123]. Patients remained intubated and sedated throughout the induction, maintenance, and rewarming phases. However, severe adverse events, including bradycardia, tachyarrhythmias, deep vein thrombosis, pulmonary oedema and cardiac arrest, resulted in premature trial termination [123].

##### Targeted Temperature Control in neonatal hypoxic-ischaemic encephalopathy (HIE)

TTC is associated with significant neurological benefits when initiated shortly after birth in neonates with HIE [124–128]. Only one study reported survival benefit from TTC [127], although it does appear to improve long-term neurocognitive function (assessed by IQ scoring at age 6–7 years) in comparison with a normothermic control group [128]. A systematic review and meta-analysis that included 11 RCTs of 1505 subjects with moderate and severe HIE showed consistently superior results with TTC in terms of mortality and neurological disability [135].

### Pharmacological modulation of metabolism

#### Hydrogen sulfphide

Hydrogen sulfphide (herein sulfphide) is a colourless gas with a rotten egg odour, historically identified as a ‘“sewer gas’” and respiratory toxicant. More recently, sulfphide was identified as an important signalling molecule with pleiotropic effects across numerous physiological systems, warranting its inclusion as the third member of the endogenous gasotransmitter class alongside nitric oxide (NO) and carbon monoxide (CO) [136,137]. In a landmark publication in 2005, Blackstone et al.. showed that exogenous sulfphide (administered as inhaled H_2_S gas) induced a “‘suspended animation-like state”’ in mice [138]. Global metabolism fell rapidly and profoundly, with a 90% fall recorded at 6 hours, as measured by whole-body calorimetry. Importantly, on cessation of the gas, animals showed no adverse effects, with neurological function similar to controls [138]. The mechanism of action is through reversible inhibition of complex IV in the mitochondrial ETC [139]. The rationale for he use of sulfphides in IRI is the decrease in mROS production generated on reperfusion by reducing mitochondrial membrane potential. As sulfphide gas would be difficult, not to mention unpleasant, to administer in a clinical environment, with potential toxicity risks to staff, attention switched to injectable sulfphide generators and donors.

#### Sulfphide generators

Sulfphide generators (e.g. sodium sulfphide and sodium hydrosulfphide; Na_2_S and NaHS, respectively) are simple salts that, upon dissolution, near-instantaneously release all of their sulfphur as sulfphide. As such, they facilitate rapid, unregulated sulfphide release, potentially causing off-target effects and compromising its putative efficacy [140]. Notwithstanding this, sulfphide generators have shown promise in various IRI animal models, including cerebral, myocardial, renal, hepatic, and global IRI [141–148]. However, on translation to patients, an RCT (NCT00858936) [149] using Na_2_S in coronary artery bypass surgery was terminated early, with results unpublished.

#### Sulfphide donors

Sulfphide donors release sulfphide in a slower, more controlled manner, influenced by several environmental factors (e.g. acidity, pH, temperature, light, and sound) and/or activation mechanisms (e.g. thiols, and enzymes) [140]. The most extensively trialled sulfphide donor in non-clinical studies, GYY4137, showed therapeutic efficacy in a model of myocardial infarction [150]. More, recently, ammonium tetrathiomolybdate (ATTM), a member of the thiometallate drug class, was identified by ourselves [140] and others [151] as a slow-release sulfphide donor. ATTM releases sulfphide in a controlled manner over time and is activated by both acidity and thiols; this dictates that more sulfphide should be released in more ischaemic, more acidic cells that would have a greater need for treatment, and, intracellularly, where higher concentrations of thiols are present [152]. Furthermore, ATTM gains intracellular access through the use of anion exchanger (AE-1) proteins that promote intracellular delivery and limit extracellular, off-target effects [152]. Administration of ATTM in animal models at the point of reperfusion conferred organ protection following myocardial infarction and stroke, and survival benefit following severe haemorrhage/resuscitation [140]. Follow-up studies in rats showed both histological and functional outcome benefits in a regional stroke model [153], and in pigs following myocardial infarction [154].

Sodium thiosulfphate (STS) had cardioprotective effects in isolated rat hearts using the Langendorff model, where it was shown to induce mitochondrial hypometabolism and enhance mROS scavenging [155,156]. Although these effects were not tested in myocardial IRI models *in vivo*, STS therapy advanced to phase II clinical trials. While a dose-–escalation study in patients with acute coronary syndrome found STS to be safe and well- tolerated [157], a subsequent phase II RCT in patients undergoing PCI for STEMI, with dosing given both pre- and 6 -hours’ post-reperfusion, found STS was well- tolerated but ineffective in reducing infarct size [158].

For more detail, readers are directed to reviews on the therapeutic effects of H_2_S and sulfphide donors in myocardial and cerebral IRI [159,160].

#### Nitric oxide

Despite being a reactive species itself, nitric oxide (NO) functions as a gaseous transmitter produced by three isoforms of nitric oxide synthase (NOS): endothelial (eNOS), neuronal (nNOS), and inducible (iNOS). iNOS is produced in response to stimuli such as inflammation and ischaemia. NO inhibits the ETC, particularly complexes I, III, and IV [69,139,161,162]. NO reversibly inhibits complex I through selective S-nitrosation of Cys39, a subunit of NADH dehydrogenase 3, a constituent protein of complex I [162]. Mechanisms underlying complex III inhibition remain unclear, while inhibition of complex IV occurs via competitive binding at the oxygen site [163].

The effects of NO on mitochondria may be pathological or beneficial, or transient or permanent, based on concentration and context. For example, the reaction of NO with superoxide generates a highly potent radical, peroxynitrite (ONOO^−^) which may lead to irreversible inhibition of the ETC [164–166]. However, NO may offer protection against IRI by activating soluble guanylate cyclase to increase the production of cyclic guanylate monophosphate and, thus, increase protein kinase. G (PKG) activity. This pathway, in turn, delays the opening of the mPTP, blocking the release of Cyt c and preventing the activation of the apoptosis [167]. NO may also delay mPTP opening through S-nitrosylation of cyclophilin, a critical mPTP mediator [168]. NO has also been shown to be an important mediator in simulating mitochondrial biogenesis [169]. Clinical trials using NO have been disappointing. In patients presenting with STEMI, inhalation of NO at 80 ppm for 4 -hours post-PCI was safe but, compared towith controls, did not impact onaffect infarct size up to 3 days post-treatment, or on left ventricular ejection fraction at 4 months [170].

#### Carbon monoxide

Carbon monoxide (CO) is a potent toxicant that impairs oxygen delivery due to its high affinity for haemoglobin, forming carboxyhaemoglobin [171], and oxygen consumption by inhibiting the ETC particularly at complex IV [139,172]. CO is, however, endogenously produced at low levels, functioning as a signalling molecule with anti-inflammatory, anti-apoptotic, and antioxidant properties [173–177]. Clinical application of CO inhalation is, however, restricted due to its potential toxicity and challenges in gas safety regulations [178,179]. Nonetheless, human safety assessments have included exposing healthy volunteers to 500 ppm CO inhalation for one1 hour, with no toxic effects reported [180]. Despite its therapeutic potential, high-dose administration of CO does remain a possible concern due to elevation of carboxyhaemoglobin levels and thus reduction in the oxygen-carrying capacity of haemoglobin [181].

To address this clinical use challenge, CO-releasing molecules (CORMs) were developed to enable safer, and better -targeted CO delivery [182–184]. In rodent models of cardiac arrest, CORMs demonstrated neuroprotective effects and improved survival [185,186]. In large animal models using extracorporeal resuscitation, CORMs producing carboxyhaemoglobin levels of 7–14% were neuroprotective [187]. In a porcine myocardial infarction model, pre-reperfusion CORM administration reduced infarct size and improved left ventricular function [188].

### Enhancing the mitochondrial antioxidant system

#### Coenzyme Q10

Ubiquinone, also known as Coenzyme Q10 (CoQ10), is both a fat-soluble electron carrier in the etc.ETcC and an exogenous mitochondrial antioxidant [189]. Its antioxidant properties arise from its redox forms (ubiquinone, semi-ubiquinone, and ubiquinol) that mitigate mROS generation by preventing premature electron interactions with oxygen. This dual role highlights the importance of CoQ10 in regulating oxidative processes, contributing to both cellular homeostasis and mitochondrial function. In acute MI models, exogenous CoQ10 regulated mitochondrial oxidative stress [190,191]. Small clinical trials have been performed in various IRI conditions, primarily elective cardiac surgery. Patients typically received CoQ10 doses for 3–5 days before surgery, a regimen not applicable to most IRI conditions,; however, the outcomes of these trials were inconsistent (Table 2).

**Table 2:** Summary of randomised controlled clinical trials employing a pharmacological approach to enhance the mitochondrial antioxidant system during ischaemia-reperfusion injury.

Study	Sample size	Condition	Treatment	Primary outcomes	Results
Tanaka et al 1982 [211]	50	Cardiac valve replacement	CoQ10	Cardiac output	Positive
Judy et al. 1993 [212]	20	CABG	CoQ10	Speed of recovery, cardiac function, ejection fraction, CoQ10 serum level	Positive
Chello et al. 1994 [213]	40	CABG	CoQ10	Arrhythmias, CoQ10 serum level, MDA, CK-MB	Positive
Taggart et al. 1996 [214]	20	CABG	CoQ10	CoQ10 serum level, troponin T, myoglobin, CK-MB	Negative
Zhou et al. 1999 [215]	24	Cardiac valve replacement	CoQ10	MDA, SOD, CK-MB	Positive
Rosenfeldt et al. 2005 [216]	121	CABG	CoQ10	CoQ10 serum level, troponin I,MDA, hospital stay, quality of life	Variable
Makhija et al. 2008 [217]	30	CABG	CoQ10	Arrhythmias, antioxidant level, inotropic need, length of stay	Variable
Mohseni et al. 2015 [218]	52	AMI	CoQ10	IL-6 level, ICAM1 level	Positive
Ramezani et al. 2020 [219]	60	AIS	CoQ10	Neurological outcomes, SOD, MDA	Negative
Dwaich et al. 2016 [220]	45	CABG	Melatonin	Troponin I, IL-1ß, Caspase-3,	Positive
Ekeloef et al. 2017 [209]	48	MI	Melatonin	Myocardial salvage index,troponin I, CK-MB, MDA, MPO	Negative
Rodrigues et al. 2016 [208]	146	MI	Melatonin	Infarct size, death, CHF	Negative
Shafiei et al. 2018 [206]	60	CABG	Melatonin	Troponin I, lactate, MDA, TNF-α	Positive
Zhao et al. 2018 [204]	60	Carotid stenosis	Melatonin	SOD, GPx, CAT, TNF-alpha, IL-6,	Positive
Panah et al. 2019 [210]	40	Renal transplant	Melatonin	Renal function, MDA, TNF-α	Positive
Nasseh et al. 2022 [207]	100	CABG	Melatonin	Troponin 1, CRP, CK-MB,length of stay	Variable
Gibson et al. 2016 [221]	118	MI	MTP131	Safety, myocardial infarct size	Safe, Negative
Saad et al. 2017 [222]	14	Renal IRI	MTP131	Renal function, BP, GFR	Positive

AIS, acute ischaemic stroke. AMI, acute myocardial infarction. BP, blood pressure. CABG, coronary artery bypass graft. CAT, catalase. CHF, congestive heart failure. CK-MB, creatine kinase myocardial band. CoQ10, coenzyme Q10. GFR, glomerular filtration rate. GPx, glutathione peroxidase. ICAM-1, intercellular adhesion molecule-1. IL-1ß, interleukin-1 beta. IL-6, interleukin-6. MDA, malondialdehyde. MI, myocardial infarction. MPO, myeloperoxidase. SOD, superoxide dismutase. TNF-α, tumor necrosis factor-alpha.

#### MitoQ (mitoquinol)

MitoQ is a modified form of CoQ10. It acts as a mitochondrial-targeted antioxidant that enters mitochondria more readily due to its positive charge. It comprises the antioxidant quinone moiety covalently attached to a lipophilic triphenylphosphonium cation [192]. Once inside the mitochondria, MitoQ accumulates on the matrix surface of the inner membrane, where it is continually recycled to the active quinol antioxidant form by complex II in the ETC [192].

MitoQ has been tested in various IRI models [193–196] but not yet in humans. Given the evidence suggesting that mitochondrial oxidative stress is a pathogenic factor in Parkinson’s disease [197,198], MitoQ has been tested in this patient group but failed to impact on disease progression [199].

#### Melatonin

Melatonin is an endogenous mitochondrial-targeted antioxidant that directly detoxifies mROS produced during IRI [200]. Experimental evidence supports its efficacy across various IRI models [201–204]. Melatonin transforms mROS into less toxic reactive species and generatinges by-products (melatonin metabolites) that also have ROS scavenging effects [205].

Two small trials found that melatonin significantly reduced myocardial damage when given for 1–5 days preoperatively in patients undergoing coronary artery bypass graft surgery [206]. In a more recent study involving 100 patients, melatonin was administered twice before reperfusion [207]. While CK-MB levels were lower in the melatonin group compared towith placebo, neither troponin nor inflammatory markers were affected. In acute STEMI patients, melatonin was given both pre- and post-reperfusion yetbut failed to show superior clinical outcomes [208,209]. Melatonin did show benefits in patients undergoing carotid endarterectomy and renal transplantation [204,210] (please refer to Table 2 for additional details). While small trials do indicate potential, the overall clinical evidence for melatonin use remains uncertain.

#### Bendavia

Bendavia, also known as MTP-131 or elamipretide, is a targeted mitochondrial antioxidant that penetrates cell membranes and selectively binds to cardiolipin in the inner mitochondrial membrane [223–225]. MTP-131 may also work by promoting mitochondrial supercomplex formation, which improves electron transfer efficiency and reduces mROS production [225,226]. MTP-131 scavenges mROS in various IRI models, including cardiac, hepatic, kidney, and cerebral [227–231]. A phase II trial in STEMI patients undergoing PCI found that MTP-131 was safe but ineffective in reducing cardiac infarct size, as measured by CK-MB [221]. In a small study of patients with renal artery stenosis undergoing revascularizsation, MTP-131 improved renal blood flow, renal function, and blood pressure stability [222]. Larger RCTs are needed for a conclusive assessment of the utility of this approach.

### Other mitochondrial-directed strategies

Various other pharmacological strategies have been tested to mitigate the damage caused by IRI through varying effects on mitochondria, though clinical data have been largely disappointing. Cyclosporine A has been investigated for its potential to prevent the opening of the mPTP, thereby blocking apoptosis and reducing mitochondrial calcium influx. While it showed promising effects in multiple experimental IRI models [232–236], these results could not be replicated in clinical trials involving patients with acute anterior ST-elevation myocardial infarction or out-of-hospital cardiac arrest [237,238].

Accumulation of the Krebs’ cycle intermediate, succinate, during ischaemia results in mROS production during reperfusion and injury. This could be blocked by dimethyl malonate, a membrane-permeable precursor of the succinate dehydrogenase competitive inhibitor, malonate [42,239–241]. A subsequent study using diacetoxymethyl malonate diester (MAM), which rapidly delivers malonate to cells, reported cardioprotectivity [242]. However, the optimal dose of dimethyl malonate remains to be defined before progression to “‘proof of concept”’ clinical studies to enhance its translational potential [242].

## Other therapeutic strategies to mitigate IRI damage

Several therapies have been trialled in large RCTs that sought to reduce calcium overload and, thus, the extent of reperfusion injury. The EXPEDITION study assessed cariporide, a sodium hydrogen exchanger isoform-1 (NHE-1) inhibitor in 5761 high-risk patients undergoing coronary artery bypass graft surgery which, by limiting intracellular sodium accumulation, also limits Na/Ca-exchanger-mediated calcium overload [243]. While there was a highly significant short-term reduction in myocardial infarction with cariporide, there was, however, an increase in mortality related to an increase in cerebrovascular events, albeit significance was lost by 6 months. Another strategy trialled in 3023 intermediate- to high-risk patients undergoing cardiac bypass graft surgery was the use of MC-1, a purinergic receptor antagonist, which prevents cellular calcium overload by antagonizsing the positive inotropic effect of extracellular ATP [244]. Unfortunately, no impact was seen on the composite outcome of cardiovascular death or nonfatal myocardial infarction.

Enthusiasm has also been shown for pre- or post-conditioning with volatile anaesthetics such as isoflurane and sevoflurane for cardio- or neuro-protection. Multiple mechanisms of action have been proposed, including impacts on adenosine signalling, activation of sarcolemmal or mitochondrial ATP-sensitive potassium channels, G protein-coupled receptor activation, and small-burst ROS production, and suppressionng of apoptosis through inhibiting the release of Ca^2+^ and phospholipase C or through activationng of Akt [245–247]. While some laboratory models, small clinical trials, and early RCTs did show benefits [245–248], these have not been reproduced in more recent large RCTs., e.g. [249,250].

## Considerations and future directions

IRI creates a state of oxidative stress responsible for organ dysfunction and activation of multiple cell death pathways. Various mechanisms are implicated, including excess mitochondrial ROS production, calcium overload, and other inflammatory processes. These are magnified during the reperfusion phase, which, thus, represents an important window for treatment. This review has focussed primarily on interventions that aim to decrease mitochondria-generated oxidative stress with a brief mention of other non-mitochondrial-targeted approaches. Unfortunately, all strategies aimed at attenuating these mechanisms have so far failed to translate to routine clinical practice, despite often promising non-clinical results and positive outcomes from small clinical cohorts, yet failure at the level of the large multi-centre randomised trial. Some reasons for these repeated failures can be identified. For example, the targeted temperature control trials did not attain target temperatures for at least 4–6 hours, by which time much of the reperfusion injury has likely occurred. Indeed, mitochondrial ROS production peaks within the first minutes of reperfusion, highlighting this period as the optimal therapeutic window for intervention [56–59]. Pre-conditioning and post-conditioning studies have adopted a wide variety of direct and remote techniques, and few, if any, head-to-head comparisons have been performed to identify an optimal strategy. Even the same concept, IPC induced by intermittent limb ischaemia, has been applied in a diverse manner. To our knowledge, no clear attempt has been made to determine the best approach. Similarly, with the pharmacological approaches, optimal dosing and duration regimens have not often not been characterised before embarking on large Phase III studies, while the Phase II studies have usually focussed on a clinical signal suggesting benefit rather than confirming a clear biological effect such as a reduction in oxidative damage. The non-clinical model designs should also come under scrutiny. These usually utilise young, healthy animals without comorbidities, and the results may not be generalisable to older, comorbid patient populations. Species differences in terms of responsible pathways and drug dosing may also be relevant.

Because of the imperative for early intervention to ameliorative oxidative stress, pharmacological approaches most likely offer the greatest chance of success, particularly for acute ischaemic conditions such as stroke, haemorrhagic shock and myocardial infarction. The main mitochondrial strategies are either to boost antioxidant defences, to prevent downstream processes such as the opening of the mPTP which can lead to cell death, or to attenuate the degree of ROS production. Our personal bias is towards the latter strategy, as this tackles the problem at the source.

A final consideration is a multi-modal approach, for example, targeting both calcium overload and mitochondrial ROS production. WhileAs an attractive proposition, this runs counter to standard approaches that rely on first demonstrating efficacy with one agent and then seeking incremental benefit from the addition of another. Until a first agent success can be clearly demonstrated, this is unlikely to happen.

## Conclusion

IRI is a complex pathological condition associated with the excess production of mROS that overwhelms endogenous antioxidant defences. The consequence is structural and functional modifications in proteins, lipids and DNA, withleading to cell death. There remains an unmet clinical need to ameliorate oxidative stress, though promising non-clinical findings have yet to translate into routine clinical practice.

## Abbreviations

ADP: adenosine diphosphate
AIS: acute ischaemic stroke
AMI: acute myocardial infarction
ATP: adenosine triphosphate
ATTM: ammonium tetrathiomolybdate
BP: blood pressure
CA: cardiac arrest (in-hospital and out-of-hospital)
CAT: catalase
CHF: congestive heart failure
CK-MB: creatine kinase myocardial band
CO: carbon monoxide
CORMs: CO-releasing molecules
CPR: cardiopulmonary resuscitation
CoQ10: coenzyme Q10
Cyt c: cytochrome c
ETC: electron transport chain
FMN: flavin mononucleotide
GFR: glomerular filtration rate
GPx: glutathione peroxidase
HIE: hypoxic-ischaemic encephalopathy
ICAM-1: intercellular adhesion molecule-1
IL-1ß: interleukin-1 beta
IL-6: interleukin-6
IPC: ischaemic pre-conditioning
IPostC: ischaemic post-conditioning
IRI: ischaemia-reperfusion injury
MAM: diacetoxymethyl malonate diester
MCAO: middle cerebral artery occlusion
MI: myocardial infarction
MPO: myeloperoxidase
M-RCT: multi-centre randomised controlled trial
NA: not applicable
NCE: sodium/calcium exchanger
NHE-1: sodium hydrogen exchanger isoform-1
NHE: sodium/hydrogen exchanger
NO: nitric oxide
NOS: nitric oxide synthase
NR: not reported
OHCA: out-of-hospital cardiac arrest
Prx: peroxiredoxin
RIPC: remote ischaemic pre-conditioning
ROS: reactive oxygen species
SOD-2: superoxide dismutase-2
SOD: superoxide dismutase
S-RCT: single-centre randomised controlled trial
STEMI: ST-elevation myocardial infarction
TNF-α: tumor necrosis factor-alpha
TRx: thioredoxin
TRxR: thioredoxin reductase
UCPs: uncoupling proteins
UQ: ubiquinone
eNOS: endothelial nitric oxide synthase
iNOS: inducible nitric oxide synthase
mPTP: mitochondrial permeability transition pore
mROS: mitochondrial reactive oxygen species
nNOS: neuronal nitric oxide synthase
